# Microwave-Assisted Synthesis of Goethite Nanoparticles Used for Removal of Cr(VI) from Aqueous Solution

**DOI:** 10.3390/ma10070783

**Published:** 2017-07-11

**Authors:** Vinh Dinh Nguyen, Jindrich Kynicky, Pavlina Ambrozova, Vojtech Adam

**Affiliations:** 1 Central European Institute of Technology, Brno University of Technology, 612 00 Brno, Czech Republic; nguyenvinhkhtn@gmail.com (V.D.N.); vojtech.adam@mendelu.cz (V.A.); 2 Faculty of Chemistry, Thai Nguyen University of Sciences, 251580 Thai Nguyen, Vietnam; 3 Department of Geology and Pedology, Mendel University in Brno, 613 00 Brno, Czech Republic; pavlina.ambroz@gmail.com; 4 Department of Chemistry and Biochemistry, Mendel University in Brno, 613 00 Brno, Czech Republic

**Keywords:** goethite nanoparticles, microwave, Cr(VI) removal

## Abstract

The microwave-assisted synthesis of goethite nanoparticles has been studied. The samples were characterized by X-ray diffraction (XRD), scanning electron microscopy (SEM), thermogravimetric analysis (TGA), differential thermal analysis (DTA) and Brunauer–Emmett–Teller (BET) method. Goethite rod-like nanoparticles have been successfully synthesized in 10 min of microwave treating at 100 °C. Particle size is in the range from 30 to 60 nm in width and from 200 to 350 nm in length. BET analysis indicated that the surface area of the product is 158.31 m^2^g^−1^. The feasibility of Cr(VI) removal fromaqueous solution depends on the pH of the solution and contact time. The maximum adsorptionis reached at pH 4.0 and 540 min of contact time. The adsorption kinetics was analyzedby the pseudo-first- and second-order models and the results reveal that the adsorption process obeys the pseudo-second-order model. The adsorption data were fitted well with the Langmuir adsorption isotherm.

## 1. Introduction

Chromium is one of the most common metals polluting the soil and water because of its applications in metal coating, tanneries, wood preservation, and pigmentation [1]. In soil and water, chromium can exist in the forms of Cr(III) and Cr(VI) in which the hexavalent state is more mobile and dangerous than trivalent one. Cr(VI) compounds resemble phosphates and sulfates and can be transported into the cell in place of these ions [2]. Cr(VI) is wellknown for causing carcinomas in animals and humans [2,3].

Cr(VI) can be removed from water by various techniques such as adsorption [4,5,6], filtration [7,8], ion exchange [9], and electrochemical treatment [10]. The adsorption technique has been wildly investigated and applied because of the variety of the sorbents and its effectiveness [10]. Iron oxides and iron oxyhydroxides are common sorbents used to remove Cr(VI) from water [9,10,11,12]. Belonging to a group of iron oxyhydroxide compounds, goethite (α-FeOOH) has been studied and applied to remove heavy metal contaminants in ground and waste water [10,11,13,14]. The ability of goethite for the removal of Cr(VI) from aqueous solution was investigated in some published articles [4,6,15,16,17], which confirmed the potential application of this kind of material for treatment of Cr(VI) in water.

Goethite can be prepared in the laboratory in various ways such as oxidation of ferrous solution [18], precipitation of ferric solution [19], or thermal transformation [20]. In the conventional preparation, the reaction time is often long from 24 h to several days and goethite particles are often in a range of several micrometers and have a low surface area [20]. Some contributions [21,22,23] revealed that when in the nanoscale, goethite particles show the high surface area and effective adsorption ability. To obtain the goethite nanoparticles, surfactants were used as the template to control the shape and size of the materials [22,23], although the process still takes a long time. The usage of microwaves to support the synthesis of nanomaterials in order to reduce the time and increase specific properties has been investigated in numerous papers [21,24,25,26,27,28,29] which suggested the ability to significantly reduce the reaction time as well as the size of the particles.

This work is aimed to investigate the using of microwaves to support the synthesis of goethite nanoparticles in order to reduce the reaction time. Moreover, the feasibility of nanogoethite for removing Cr(VI) from aqueous solution has been estimated and the isotherm of adsorption process has been studied.

## 2. Materials and Methods

### 2.1. Reagents

All reagents used were analytical grade. K_2_CrO_4_, HCl, NaOH, Fe(NO_3_)_3_·9H_2_O and NaNO_3_ were supplied by Merck (Darmstadt, Germany) and used without further purification. All the solutions were prepared using deionized water.

### 2.2. Synthesis of Goethite Nanoparticles

To investigate the effect of the microwave process on the synthesis of goethite nanoparticles, four samples were prepared according to the following procedure: in each experiment, 4.04 g of Fe(NO_3_)_3_·9H_2_O was dissolved in 100 mL of distilled water through vigorous stirring. pH of the solution was adjusted to 12 by 1 M NaOH solution and the mixture was constantly stirred for 30 min. The obtained suspension was transferred into a 500 mL round-bottom flask and heated in a microwave oven at 100 °C for 5, 10, 20 and 30 min, respectively. While heating, a water condenser was employed to prevent the loss of water from the reaction system. The obtained yellowish-brown solid was separated from the supernatant solution by using a centrifuge and washed several times with a solution of distilled water and ethanol (ratio 1:1), then dried at 100 °C for 24 h.

### 2.3. Characterization of the Sample

The phase of the obtained powder was characterized by XRD using aD8 Advance diffractometer (Bruker, Madison, WI, USA) with CuKα radiation (λ = 1.5406 Å), in the 2θ scale range from 20° to 70° with the step of 0.02°. The morphology was investigated by scanning electron microscopy (S4800 Hitachi, Tokyo, Japan). TGA and differential thermal analysis (DTA) curves of the sample were recorded on a Labsys Evo S60/58988 instrument (Setaram, Caluire, France) in the 50–800 °C range, with the temperature rate of 10 °C·min^−1^. The surface area of the sample was determined by the Brunauer–Emmett–Teller (BET) method on a Micromeritics Tristar 3000 apparatus (Micromeritics, Aachen, Germany). 

### 2.4. Cr(VI) Removal Study

The sorption experiments were carried out in stopper conical flasks placed in a bath shaker at room temperature. To optimize the adsorption process, the specific parameters such as pH, equilibration time, and initial concentration of Cr(VI) ion influencing the adsorption process have been investigated by changing one parameter and keeping other parameters constant. In each experiment, 0.2 g of goethite was added to a solution containing NaNO_3_ 0.05 M, Cr(VI) ion with a definite concentration, and at a specific pH. The mixture had a volume of 100 mL and was shaken for the desired time at the rate of 150 rpm. Then, the solid was separated by the centrifuging and filtering. The concentration of Cr(VI) in the filtrate was analyzed using atomic absorption spectroscopy (Thermo M Series, Waltham, MA, USA). The experiments were carried out in duplicate and mean values were presented. The error obtained was 1–2.0%. The data were reported in terms of removal efficiency (*E*) and the amount of adsorbate/weight of adsorbent at equilibrium (*q*_e_).

*E*(%) = (*C*_o_ − *C*_e_) · 100/*C*_o_, (1)

*q*_e_ = (*C*_o_ − *C*_e_) · *V*/*w*, (2)

where *C*_o_ and *C*_e_ are initial and equilibrium Cr(VI) concentration of the solutions (µM), *V* is the volume of the solution (L), *w* is the weight of adsorbent (g). 

For the isotherm study, the initial concentration of Cr(VI) was varied from 10 to 150 µM. To describe the adsorption process between solid–liquid interfaces, the data was fitted to the Langmuirand Freundlich (4) adsorption models [9,30,31], respectively:

*q*_e_ = *C*_e_ · *q*_m_ · *K*_L_/(1 + *C*_e_ · *K*_L_), (3)

*q*_e_ = *K*_F_ · *C*_e_^1/n^ (4)

where *q*_m_ is the maximum adsorption, *K*_L_ is the Langmuir coefficient, *K*_F_ and n are constants of the Freundlich model.

In addition, the feasibility of adsorption process is estimated by a constant R_L_ [31] which can be calculated as:

R_L_ = 1/(1 + *C*_o(max)_ · *K*_L_), (5)

where *C*_o(max)_ is the highest initial adsorbate concentration.

## 3. Results and Discussion

### 3.1. Chemical and Physical Properties of Nanoparticles

**Figure 1:**
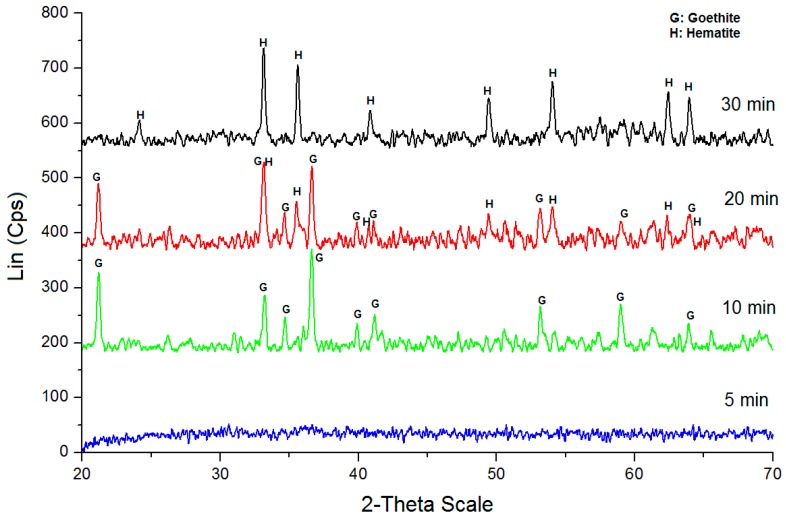
XRD patterns of the samples obtained at different times.

The X-ray diffraction patterns of the powders obtained with different durations of the microwave processing were presented in Figure 1.

**Figure 2:**
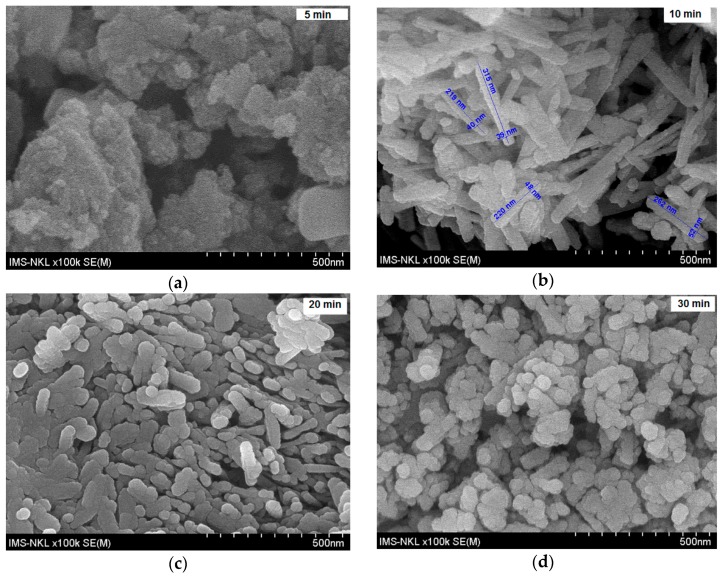
SEM images of the powders obtained in 5 min (**a**); 10 min (**b**); 20 min (**c**); and 30 min (**d**).

In the XRD pattern of the sample formed in 5 min, there cannot be found any particular diffraction peaks, indicating that this sample exists in an amorphous phase. The SEM image (Figure 2) of this sample displays the particles with an irregular shape and size. The XRD pattern of the sample obtained at 10 min exhibits the diffraction peaks at 2θ angles 21.29, 33.29, 34.73, 36.70 and 53.3 degree corresponding with the reflection of planes (101), (301), (210), (111) and (212), respectively, in the crystal structure of pure orthorhombic α-FeOOH (JCPDS 29-0713) [20] with lattice constants of a = 4.608 Å; b = 9.956 Å and c = 3.0215 Å and space group Pb nm [20]. The diffraction peaks attributed to other phases are not observed, indicating that the product contains single crystalline goethite. The SEM image of this sample shows the rod-like particles in accordance with the crystal morphology of α-FeOOH. 

The particle size varies from 200 to 350 nm in length and from 30 to 60 nm in width. The XRD pattern of the sample synthesized in 20 min presents the typical diffraction peaks of goethite. Here, however, an additional XRD pattern appears due to the hematite phase, implying that hematite is a by-product along with goethite. The SEM image of this powder shows both rod-like particles of goethite and spherical particles of hematite. On the diffractogram of the sample obtained at 30 min, the diffractions peaks of hematite become higher and those of goethite are not detected, indicating that this sample contains only the hematite phase. The spherical particles of hematite can be clearly observed in the SEM image.

**Figure 3:**
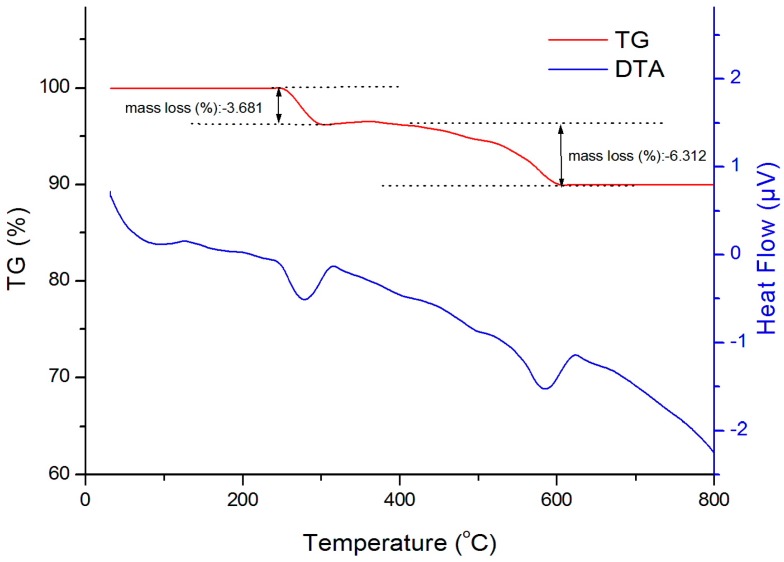
Thermal analysis diagram of the sample obtained in 10 min.

Figure 3 presents the thermal gravimetric analysis (TGA) and the differential thermal analysis (DTA) curves. From room temperature to 800 °C, there are two endothermic peaks in the ranges from 250 to 320 °C and from 550 to 620 °C on the DTA curve, respectively, corresponding to two weight loss effects on the DTA curve [20]. The reason for this can be the thermal decomposition of the goethite phase to form the hematite phase. The total weight loss is 9.99% while this value theoretically calculated is 10.12%, implying that the obtained powder is nearly pure α-FeOOH.

The surface area and pore volume of the obtained goethite determined by the BET method are 158.31 m^2^g^−1^ and 0.07 cm^3^g^−1^, respectively, while the surface area of that obtained by the conventional method is in the range from 8 to 100 m^2^g^−1^ [20,32].

The results indicated that with the support of microwave, goethite nanoparticles with a high surface area and high purity can be successfully synthesized in a short time at 100 °C under normal pressure. This suggests an effective method for synthesizing goethite.

### 3.2. Cr(VI) Removal Feasibility

#### 3.2.1. The Effect of Solution pH

**Figure 4:**
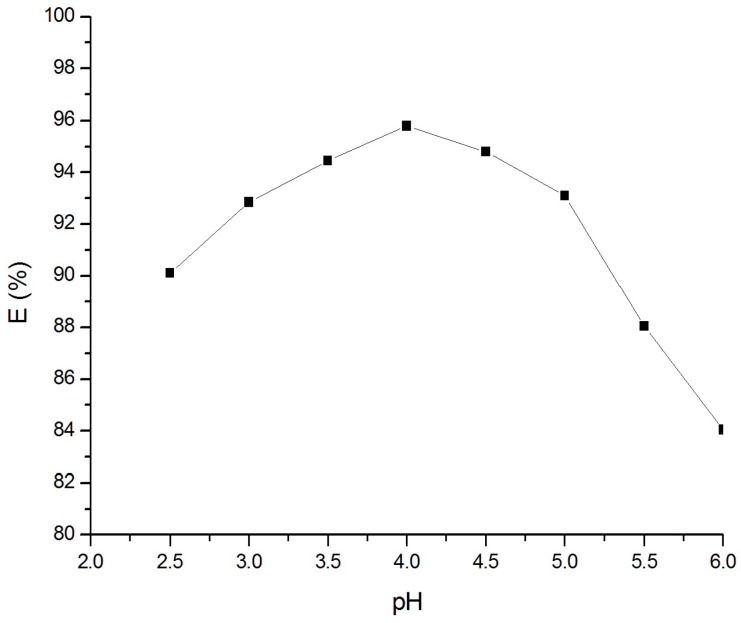
Adsorption of Cr(VI) on goethite at different pH values.

To find out the optimum pH for Cr(VI) adsorption on goethite, experiments were carried out at different pH values, from 2.5 to 6.0 with the Cr(VI) concentration of 80 µM in 600 min. The results (Figure 4) indicate a noticeable effect of pH on the adsorption of Cr(VI) on goethite. Namely, the removal efficiency of Cr(VI) increases from 90.10% to 95.80% when increasing the pH from 2.5 to 4.0 and then decreases when the pH is above 4.0.

This may be due to the fact that the adsorption of Cr(VI) on goethite is a surface complexation reaction between Cr(VI) and goethite surface [33]. The feasibility of contaminant removal of goethite noticeable depends on the pH of the solution because the charge of the goethite surface can change at the following equilibrium:

=FeOH + H^+^ ⇆ FeOH^2+^, (6)

=FeOH ⇆ FeO^−^ + H^+^. (7)

The Equilibrium (6) is dominant and the surface is positively charged when the pH of the solution is below pH pzc (the point of zero charge), generally from 7.5 to 9.0. In contrast, the surface is negatively charged [20,34]. It means that the affinity of goethite to anion increases when the pH decreases. However, the transforming of Cr(VI) in aqueous solution is complicated because, at a low pH (below 1.0), Cr(VI) dominantly exist in the form of H_2_CrO_4_, while at a higher pH, it can exist in the form of HCrO_4_^−^, CrO_4_^2−^, Cr_2_O_7_^2−^ etc. depending on the pH value and concentration [33,35]. It can be supposed that the pH value of 4.0 is the optimum value for interaction between Cr(VI) ions and the goethite surface.

#### 3.2.2. Effect of the Contact Time on the Cr(VI) Removal

**Figure 5:**
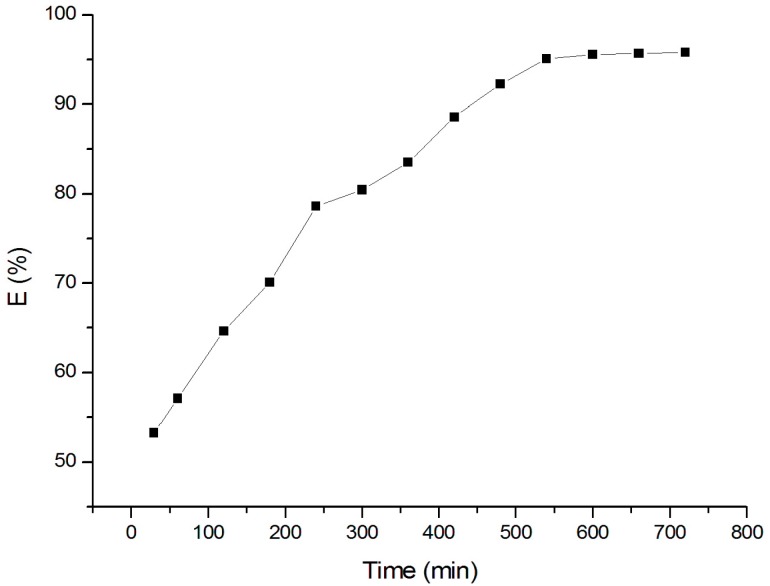
Adsorption of Cr(VI) on goethite with different contact times.

The study for Cr(VI) adsorption on goethite has been performed at different times from 30 to 720 min with Cr(VI) concentration of 80 µM at pH 4.0 to find the optimum adsorption contact time. The adsorption data (Figure 5) reveals that the amount of Cr(VI) sorbed by goethite increases with increasing contact time until the equilibrium is reached. The adsorption of Cr(VI) on goethite is 57% at 30 min of contact time, then regularly increases until it reaches around 95% at 540 min. The adsorption nearly remains stable after 540 min of contact time. The obtained data suggests that 540 min of contact time is sufficient for the Cr(VI) adsorption on goethite to attain equilibrium.

To study the kinetics of Cr(VI) adsorption on goethite, the data were analyzed by using pseudo-first- and second-order models [36,37]:

log(*q*_e_ − *q*_t_) = −*k*_1_*t*/2.303 + log*q*_e_, (8)

*t*/*q*_t_ = *t*/*q*_e_ + 1/(*k*_2_ × *q*_e_^2^), (9)

where *t* is the time (min), *q*_t_ (µmol g^−1^) is the amount of Cr(VI) adsorbed at the time *t*; *k*_1_ (L min^−1^) and *k*_2_ (g min^−1^ µmol^−1^) are adsorption rate constants of the pseudo-first-order model and pseudo-second-order model, respectively. Results were given in Table 1.

The correlation coefficients R^2^ of the pseudo-second-order kinetic model for Cr(VI) adsorption is 0.992, higher than that of the pseudo-first-order kinetic model. Moreover, the *q*_e_ value calculated from the pseudo-second-order model is better in agreement with the experimental data when compared to that of the pseudo-first-order model. Therefore, it can be assumed that the adsorption kinetics of Cr(VI) on goethite obey the pseudo-second-order kinetic model.

#### 3.2.3. Adsorption Isotherm

**Figure 6:**
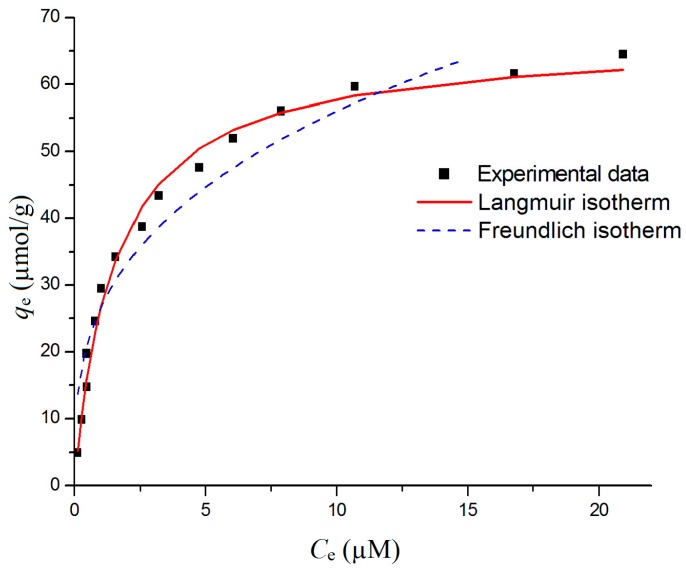
Langmuir and Freundlich isotherms for Cr(VI) adsorption on goethite.

Initial concentration and the maximum capacity of adsorption are important parameters for Cr(VI) removal. In order to predict the adsorption trend and the maximum amount of Cr(VI) sorbed on goethite, the adsorption experiments have been carried out with different initial concentrations of Cr(VI) at a pH of 4.0 and for 540 min and the results were fitted to the Langmuir and Freundlich models (using OriginLab software, 9.0, OriginLab Corporation, Northampton, MA, USA). Results were presented in Figure 6 and Table 2.

The correlation coefficient, R^2^ of two models is above 0.9 which confirms that the adsorption data fit well to both models. *K*_L_ calculated from the Langmuir model is 0.651 and n obtained from the Freundlich model is 3.076, implying that goethite has a high affinity for Cr(VI) adsorption. However, R^2^ of the Langmuir model is 0.989, higher than that of the Freundlich model, indicating that the data is better in agreement with the Langmuir model.

In addition, R_L_ calculated according to (5) is 0.010, suggesting that the adsorption is highly favorable. Based on the Langmuir model, the predicted maximum capacity adsorption is 66.735 µmol g^−1^, demonstrating the high feasibility of Cr(VI) removal using goethite.

## 4. Conclusions

Goethite nanoparticles have been successfully synthesized in a short time; 10 min, with the assistance of the microwave. The XRD and TGA data indicated that the obtained powder contains pure goethite. Goethite nanoparticles have rod-like morphology with the length of 200–350 nm and the width of 30–60 nm. The surface area and the pore volume of the product prepared with the assistance of the microwave are higher than that of the product obtained in the conventional way. This shows the potential application of the microwave for synthesizing of goethite.

The adsorption depends on the solution pH and contact time, obeys the pseudo-second-order model, and fits well to the Langmuir model. The optimum condition for Cr(VI) adsorption on goethite is a pH of 4.0, contact time of 540 min and the maximum adsorption is 66.735 µmol g^−1^. Goethite nanoparticles show a high feasibility of Cr(VI) removal.

## Figures and Tables

**Table 1:** Kinetic parameters of two models.

Kinetic Model	Parameters		
Pseudo-first-order	*q*_e_ (µmol g^−1^)	*k*_1_ · 10^−3^ (min^−1^)	R^2^
	46.633	8.982	0.877
Pseudo-second-order	*q*_e_ (µmol g^−1^)	*k*_2_ · 10^−4^ (g µmol^−1^min^−1^)	R^2^
	41.667	3.630	0.992
Experimental value	*q*_e(experiment)_ = 38.328 µmol g^−1^		

**Table 2:** Parameters calculated for Langmuir and Freundlich models.

Model	Parameters		
Langmuir	*q*_m_ (µmol g^−1^)	*K*_L_	R^2^
	66.735	0.651	0.989
Freundlich	N	*K*_F_	R^2^
	3.076	28.782	0.927
